# Genomic Description of ‘*Candidatus* Abyssubacteria,’ a Novel Subsurface Lineage Within the Candidate Phylum Hydrogenedentes

**DOI:** 10.3389/fmicb.2018.01993

**Published:** 2018-08-28

**Authors:** Lily Momper, Heidi S. Aronson, Jan P. Amend

**Affiliations:** ^1^Department of Earth, Atmospheric and Planetary Sciences, Massachusetts Institute of Technology, Cambridge, MA, United States; ^2^Department of Biological Sciences, University of Southern California, Los Angeles, CA, United States; ^3^Department of Earth Sciences, University of Southern California, Los Angeles, CA, United States

**Keywords:** subsurface biosphere, metagenomics, microbial dark matter, Abyssubacteria, Hydrogenedentes

## Abstract

The subsurface biosphere is a massive repository of fixed carbon, harboring approximately 90% of Earth’s microbial biomass. These microbial communities drive transformations central to Earth’s biogeochemical cycles. However, there is still much we do not understand about how complex subterranean microbial communities survive and how they interact with these cycles. Recent metagenomic investigation of deeply circulating terrestrial subsurface fluids revealed the presence of several novel lineages of bacteria. In one particular example, phylogenomic analyses do not converge on any one previously identified taxon; here we describe the first full genomic sequences of a new bacterial lineage within the candidate phylum Hydrogenedentes, ‘*Candidatus* Abyssubacteria.’ A global survey revealed that members of this proposed lineage are widely distributed in both marine and terrestrial subsurface environments, but their physiological and ecological roles have remained unexplored. Two high quality metagenome assembled genomes (SURF_5: 97%, 4%; SURF_17: 91% and 4% completeness and contamination, respectively) were reconstructed from fluids collected 1.5 kilometers below surface in the former Homestake gold mine—now the Sanford Underground Research Facility (SURF)—in Lead, South Dakota, United States. Metabolic reconstruction suggests versatile metabolic capability, including possible nitrogen reduction, sulfite oxidation, sulfate reduction and homoacetogenesis. This first glimpse into the metabolic capabilities of these cosmopolitan bacteria suggests that they are involved in key geochemical processes, including sulfur, nitrogen, and carbon cycling, and that they are adapted to survival in the dark, often anoxic, subsurface biosphere.

## Introduction

Although the subsurface biosphere is devoid of light and is often extremely carbon and energy-limited, it is home to the vast majority of Earth’s microbes, as much as 90% by the most recent estimates (Whitman et al., 1998; Kallmeyer et al., 2012; Mcmahon and Parnell, 2014; Parkes et al., 2014; Bar-On et al., 2018). Microbial metabolism is critical to the fixation, maintenance and turnover of carbon, nitrogen, and sulfur reservoirs in the subsurface biosphere. Recent studies have shown that a high proportion of the organisms in the subsurface have yet to be cultured, known only by culture-independent genetic evidence such as metagenomic shotgun sequencing of whole genomic DNA (Wrighton et al., 2012; Castelle et al., 2015). In addition, newly identified microbial lineages are now commonly implicated in major geochemical cycles (Rasigraf et al., 2014; Baker et al., 2016). It follows that there is substantial metabolic diversity yet to be discovered within the marine and terrestrial subsurface ecosystems. To better describe and model the microbial networks that catalyze carbon, nitrogen, and sulfur cycling in the subsurface, we need to incorporate newly identified microbial lineages along with their metabolic capabilities.

Molecular environmental surveys have provided a wealth of data elucidating microbial phylogenetic diversity, especially in previously poorly sampled habitats. Sequencing of small-subunit ribosomal RNA (SSU rRNA) genes directly from the environment has vastly expanded our knowledge of the microbial tree of life (Pace, 2009; Yarza et al., 2014; Probst et al., 2018). Advances in cultivation-independent methods for examining uncultured microbes, including single-cell genomics and deep sequencing of environmental samples (metagenomics), have begun yielding complete or near-complete genomes from many novel lineages (Iverson et al., 2012; Kantor et al., 2013; Brown et al., 2015; Castelle et al., 2015; Sekiguchi et al., 2015; Anantharaman et al., 2016; Momper et al., 2017a). These candidate lineages, previously recognized only through SSU rRNA sequencing and for which we have no cultured representatives, are providing a more complete view of the tree of life and a better understanding of global microbial ecology (Rinke et al., 2013; Hug et al., 2016; Parks et al., 2017). A few of the recently discovered and thoroughly described candidate phyla include the Korarchaeota (Elkins et al., 2008), Hadesarchaea (Baker et al., 2016) Kryptonia (Eloe-Fadrosh et al., 2016), Woesarchaetoa and Pacearchaeota (Castelle et al., 2015). However, in many cases, an in-depth analysis of the metabolic potential and environmental interaction of these newly discovered lineages is lacking (Parks et al., 2017; Tully et al., 2017a,b) In order to grasp the environmental relevance of lineages that are not yet cultured, we must conduct comprehensive evaluations of their metabolic potential, taking into consideration the environments and geochemical conditions in which they survive.

To that end, we present the phylogenetic and metabolic analysis of the first two genomes of a novel bacterial lineage that was recently identified in the deep terrestrial subsurface (Momper et al., 2017a). According to 16S rRNA and phylogenomic analyses, we determined that the metagenome assembled genomes (MAGs), labeled as SURF_5 and _17, constitute the first full genomes of a novel lineage. We follow proposed standards (Konstantinidis et al., 2017) to characterize this uncultivated candidate lineage and name it ‘*Candidatus* Abyssubacteria’. *Candidatus* applies both to the organism as well as the potential new lineage. This allows us to combine environmentally derived genome sequence taxonomic classification with currently accepted nomenclature standards, as proposed by Hedlund et al. (2015). In this study, we use full genome sequencing and metabolic reconstruction to elucidate the probable ecological importance of this newly identified candidate lineage. We show that members of this lineage are globally distributed in terrestrial and marine subsurface environments and possess putative functional adaptations that enable them to thrive in these dark, often anoxic environments. We use pairwise average nucleotide and average amino acid identities (ANI/AAI) to assess the taxonomic rank classification, and reconstructions based on concatenated ribosomal protein sequences to understand its relatedness within the broader scope of bacterial phylogeny.

## Materials and Methods

### Sample Collection, DNA Extraction and Sequencing

All fluid samples and corresponding geochemical data were collected from the former Homestake gold mine (now Sanford Underground Research Facility, SURF) near Lead, South Dakota, United States (44°21′ N 103°45′ W) in October, 2013. Geochemical measurements were made either on site, or upon return to the laboratory (Osburn et al., 2014). In this study, we examined deep fracture fluids from legacy boreholes drilled ∼1.5 kilometers below surface (kmbs), accessing a deeply circulating terrestrial aquifer. A comprehensive description of the fluid sampling methods can be found in Osburn et al. (2014) and Momper et al. (2017b). In brief, the biomass in borehole fluids was collected on 47 mm, 0.2 μm Supor filters (Pall Corporation, Port Washington, NY, United States). These filters were then stored on dry ice, transported to the University of Southern California and immediately frozen at -80°C. Whole genomic DNA was extracted using a modified phenol-chloroform extraction with ethanol precipitation as previously described in Osburn et al. (2014). DNA concentration was checked on a Qubit 2.0 fluorometer (Thermo Fisher Scientific), and purity was measured on a NanoDrop 2000 spectrophotometer (Thermo Fisher Scientific) before samples were sent for sequencing. Sequencing was performed at the University of Southern California’s Genome and Cytometry Core Facility (Los Angeles, United States) on an Illumina HiSeq 2500 (San Diego, CA, United States) using a paired-end method, insert size of 500 base pairs (bp) and fragment size of 150 bp, as described in Momper et al. (2017a).

### *De novo* Assembly and Read Mapping

Reads were quality trimmed and filtered using Trimmomatic version 0.36, with a minimum quality score of 40 and a minimum length of 36 base pairs (Bolger et al., 2014). Reads were then assembled using IDBA-UD 1.1.1 (Peng et al., 2012). Sequences from both fluid samples were co-assembled in order to implement differential coverage binning methods for genome bin analysis. Minimum contig length for the co-assembly was set at 10,000 bp. Coverage information was then attained by individually mapping the paired-end reads of each of the two samples to this co-assembly using Bowtie2 (Langmead and Salzberg, 2012). To convert alignments to the SAM format, the BWA-SAMPE algorithm was used with default parameters. Coverage information was extracted using SAMtools 0.1.17 (Li et al., 2009).

### Metagenome Assembled Genome (MAG) Reconstruction

Individual MAGs were reconstructed using sequence composition, differential coverage and read-pair linkage through the CONCOCT program (Alneberg et al., 2014). MAGs were then manually refined and curated using the interactive interface in the Anvi’o program (Eren et al., 2015). After refinement, genome bin completeness and contamination were re-calculated using five widely accepted marker gene suites compiled from Creevey et al. (2011), Dupont et al. (2012), Wu and Scott (2012), Campbell et al. (2013), and Alneberg et al. (2014). Reconstruction and identification of 16S rRNA gene sequences within each bin was completed using the CheckM pipeline (Parks et al., 2015). All requisite code for calculating completeness and contamination and identifying 16S rRNA sequences can be found at https://github.com/Ecogenomics/CheckM. The 16S rRNA gene sequence recovered from SURF_17 was compared to existing isolates and environmental clone sequences using the SILVA Incremental Aligner (Pruesse et al., 2012). It was also compared to existing isolates and environmental clone sequences using the NCBI Basic Local Alignment Search Tool (BLAST), querying both the ref_seq and nr databases. Closest neighbors generated from these searches were used to generate a 16S phylogenetic species tree (**Supplementary Figure 1**). The 16S rRNA gene sequence could not be recovered from SURF_5 so it was not included in this portion of the analysis.

### Phylogenomic Analyses and Phylogenetic Classification

A phylogenetic tree was constructed using a concatenation of 16 syntenic and co-located highly conserved ribosomal proteins according to Hug et al., 2016. A full list of all proteins used to build the phylogenetic tree can be found in **Supplementary Data File 1**. Ribosomal proteins were extracted from SURF_5 and SURF_17 genomes and ∼18,000 publicly available environmental genomes using Prodigal and HMMER hmmsearch (Hyatt et al., 2010; Finn et al., 2011) and were aligned with reference proteins using the MUSCLE aligner (Edgar, 2004a,b; Graham and Tully, 2018). The concatenated alignment was trimmed using TrimAl (parameter -automated1) and used to build a comprehensive phylogenetic tree using FastTree with gamma and lg parameters (Capella-Gutiérrez et al., 2009; Price et al., 2009, 2010). This comprehensive tree containing ∼18,000 genomes was then culled to include a distribution from all available bacterial phyla. All available genomes from the phylum ‘*Ca.* Hydrogenedentes’ were kept in the phylogenomic tree. The culled tree was then rebuilt using the RAxML maximum likelihood method with the GTR model of nucleotide substitution under the gamma- and invariable- models of rate heterogeneity (Stamatakis, 2006).

Pairwise average nucleotide identity (ANI) and average amino acid identity (AAI) were calculated for the SURF_5 and SURF_17 genomes against the three most closely related available genomes from the ‘*Ca.* Hydrogenedentes’ phylum. ANI and AAI were analyzed using the publicly available tools provided through the ChunLab online Average Nucleotide Identity Calculator (Yoon et al., 2017), and the CompareM amino acid identity workflow (Parks, 2014), respectively. The ANI calculator estimates the average nucleotide identity using both best hits (one-way ANI) and reciprocal best hits (two-way ANI) between two genomic datasets (Goris et al., 2007). ANI values between genomes of the same species are >95%. ANI values <75% are not reliable (Rodriguez-R and Konstantinidis, 2014). In cases of low ANI values, AAI values were also calculated and reported. The percentage of conserved proteins (POCP) between the two SURF genomes was calculated via a pairwise BLAST of SURF_5 and SURF_17 protein sequences and calculations were performed as outlined by Qin et al. (2014).

## Results

### MAG Statistics and Identification of a Novel Bacterial Candidate Phylum

**FIGURE 1:**
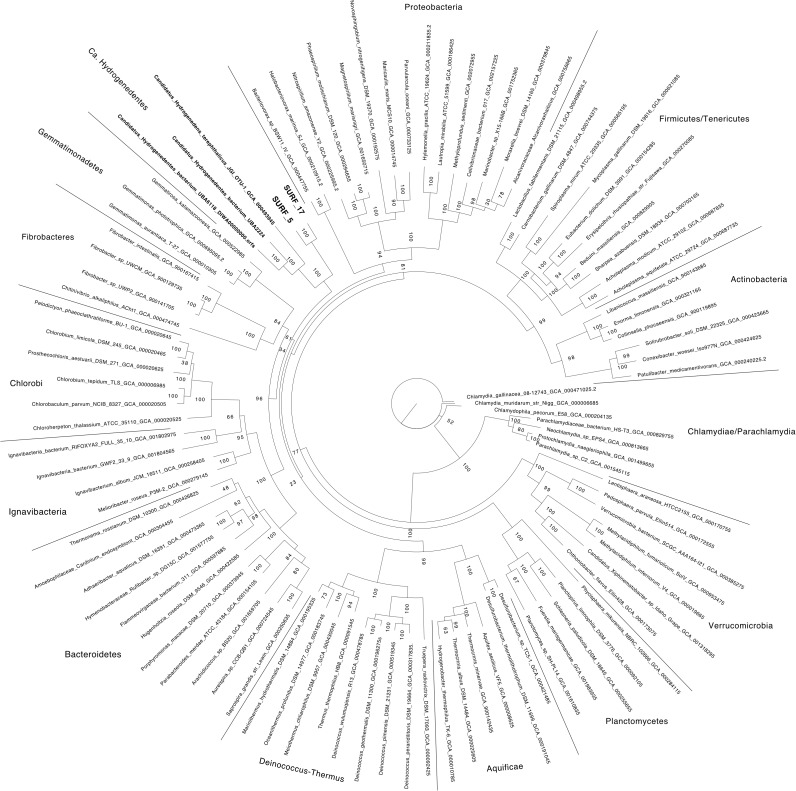
Bacterial phylogeny estimated from the maximum likelihood phylogeny of 16 concatenated ribosomal proteins is shown. SURF_5 and SURF_17 collected from SURF fluids are indicated with bold-face font. Scale bar indicates 0.2 amino acid substitutions. Support values are reported from 1000 replicates, with a value of ‘100’ shortened to ‘1’ for brevity. The list of proteins used to build the tree can be found in **Supplementary Data File 1**.

Reassembly of the metagenomic data combined with differential-coverage based binning methods yielded near-complete recovery of two novel, distinct genomes: SURF_5 and SURF_17 (97 and 91% completeness, respectively, both with 4% contamination). According to recent published standards (Bowers et al., 2017), the two MAGs reported here are high quality genomes, with greater than 90% completeness and less than 5% contamination (**Table 1**). BLAST results of the 16S rRNA gene sequence from SURF_17 against the NCBI nr and ref_seq databases revealed 80–83% sequence identity to cultured isolates of the Deltaproteobacteria, Gammaproteobacteria and Firmicutes, with no consensus converging on any one of those classes or phyla. A species tree of the 16S rRNA gene sequence shows that SURF_17 putatively belongs to an uncultured group of bacteria related to the phylum Poribacteria (**Supplementary Figure 1**). Additional phylogenomic analysis using 16 concatenated ribosomal proteins showed that SURF_5 and _17 genomes are likely members of the Candidate phylum ‘Hydrogenedentes’ (**Figure 1**). The three most closely related genomes, according to ribosomal protein sequence similarity, are the sole members of a novel candidate phylum: Candidatus Hydrogenedens terephthalicus_JGI_OTU1, Candidatus Hydrogenedentes UBA2224 and Candidatus Hydrogenedentes UBA6118 (**Figure 1**). This difference in phylogenetic placement between the single 16S rRNA gene and a concatenation of ribosomal proteins illustrates the limited reliability of reconstructed 16S sequences alone. Indeed, previous studies have noted the discrepancy between the percent identity of partial or reconstructed 16S rRNA sequences versus phylogeny based on full length 16S or multiple concatenated ribosomal sequences (Schloss, 2010; Parks et al., 2017). In this study, we deem the phylogeny based on multiple ribosomal proteins to be more robust, and will base our discussion of SURF_5 and _17 genomes on the phylogeny displayed in **Figure 1**.

**Table 1:** General characteristics of metagenome assembled genomes analyzed in this study.

	SURF_5	SURF_17
Completeness (%)	96	91
Contamination (%)	4	4
GC (%)	54.7	54.8
Genome size (Mbp)	5.1	4.6
Number scaffolds	145	144
Longest scaffold (bp)	208,377	111,957
Number of genes	4482	4105
Fraction unclassified genes	0.60	0.60
16S rRNA gene? (y/n)	n	y
Collection temperature C	22	22
Collection depth (mbs)	1,500	1,500


*Mbp, million base pairs*

*mbs, meters below surface*

Further whole genome comparison at the nucleotide and amino acid sequence levels consistently indicated that SURF_5 and SURF_17 were too divergent from the three previously sequenced ‘*Ca.* Hydrogenedentes’ genomes to have meaningful ANI (**Supplementary Data File 2**). The ANI values between ‘*Ca.* Abyssubacteria’ and other similar genomes were all below 70% and hence were not reliable for direct comparison (Rodriguez-R and Konstantinidis, 2014). The AAI values between ‘*Ca.* Abyssubacteria’ and other genomes within the ‘*Ca.* Hydrogenedentes’ phylum were all in the 45–55% range (**Supplementary Data File 2**), a relatively low value that could indicate the SURF genomes represent a novel phylum or order (Luo et al., 2014), although the undersampling and lack of available genomes from the candidate phylum Hydrogenedentes makes it difficult to determine the taxonomic level represented by the SURF genomes with confidence. Genomes SURF_5 and SURF_17 were also compared to each other. The AAI value between these two genomes was ∼65%, and the POCP value was 67%, indicating that these two genomes belong to the same family or genus-level taxonomic classification (Konstantinidis and Tiedje, 2005; Qin et al., 2014).

### Metabolic Reconstruction

**FIGURE 2:**
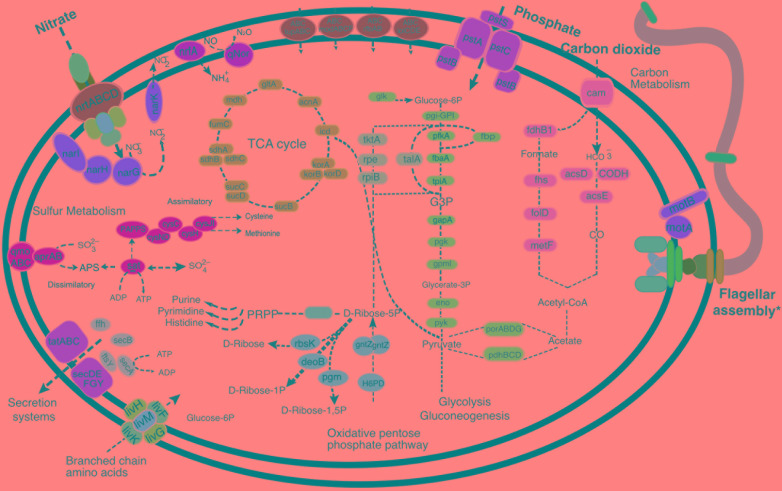
Metabolic reconstruction of the typical “*Candidatus* Abyssubacteria” cell. Key metabolic predictions and novel features identified in “*Ca.* Abyssubacteria” genomes, with full gene information available in **Supplementary Data File 3**. ^∗^Full annotations and machinery for motility and flagellar assembly can be found in **Supplementary Figure 2**.

**FIGURE 3:**
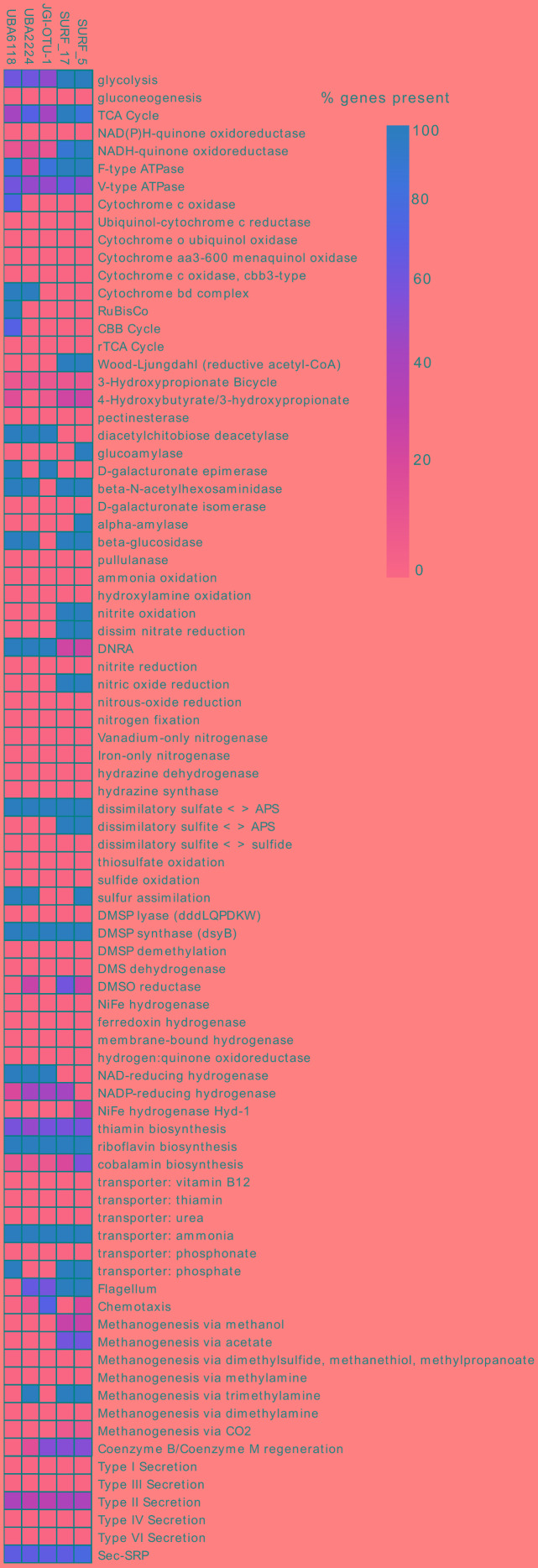
Metabolic comparison between SURF genomes and the three most closely related available genomes, from the candidate phylum ‘Hydrogenedentes’. Completeness of a given pathway or metabolism is calculated by identifying the requisite genes for that pathway that are not involved in other cellular processes, and calculating the percentage of those genes that are present in each of the five genomes. Darker red boxes indicate a larger percentage of requisite genes are present, with decreasing percentages represented by lighter shades. Complete list of genes and code used to generate the heatmap can be found at https://github.com/bjtully/BioData/blob/master/KEGGDecoder/KOALA_definitions.txt.

The availability of two nearly complete ‘*Ca.* Abyssubacteria’ MAGs enabled metabolic and putative functional predictions for this novel candidate lineage (**Figures 2, 3**). ‘*Ca.* Abyssubacteria’ contains genes that encode proteins for a complete tricarboxylic acid (TCA) cycle and all key metabolic enzymes for Embden–Meyerhof glycolysis and the pentose phosphate pathways. In both ‘*Ca.* Abyssubacteria’ genomes we found genes for the complete reductive acetyl-CoA carbon fixation (Wood-Ljungdahl) pathway (**Figures 2, 3** and **Supplementary Data File 3**) and multiple copies of the carbon dioxide transporter, carbonic anhydrase, were found in both genomes, possibly facilitating import of carbon dioxide gas into the cell for subsequent fixation. Genes for nitrate transport (*ntrABCD*) into the cell and subsequent chemotrophic nitrate reduction (*narIHGK*) were also identified. The SURF_5 genome contains the canonical gene for dissimilatory nitrate reduction to ammonium (DNRA, *nrfA*) and both genomes are putatively capable of nitric oxide reduction to nitrous oxide (qNOR). Pathways for both assimilatory and dissimilatory sulfur metabolisms were identified. We also found abundant metal and polysaccharide transporters, including those for molybdate (*modABCM*), tungstate (*tupABC*), lipoproteins (*lolCDE*), and lipopolysaccharides (*rfbAB*). The genomes from SURF do not contain complete gene sets for any of the six well characterized secretion systems (**Figure 2**), but contain a mostly complete Sec-SRP system consisting of *secABDEFGY* (**Figures 2, 3**). ‘*Ca.* Abyssubacteria’ is a motile bacterium with putative chemotaxis proteins. An enlarged version of the flagellar assembly, chemotaxis cassette and complete gene annotations can be found in **Supplementary Figure 2** and **Supplementary Data File 3**.

## Discussion

### Distribution, Habitat and Phylogeny of ‘*Candidatus* Abyssubacteria’

**FIGURE 4:**
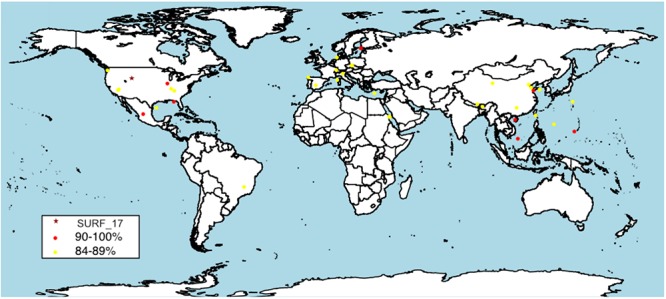
Global distribution of SURF_5 and SURF_17 relatives as measured by 16S rRNA clone nucleotide identity. The reconstructed SURF_17 16S gene was used as query sequence in the NCBI nr database. Top 50 results were included, results range from 98 to 84% identity. A complete list of 16S gene results, including accession numbers, query coverage, identity and collection source can be found in **Supplementary Data File 4**.

A BLAST search of the NCBI nr database using the 16S rRNA gene sequence from SURF_17 revealed that members of the proposed candidate lineage, ‘*Ca.* Abyssubacteria’, are globally distributed (**Figure 4**) in marine and terrestrial, shallow and deep, subsurface environments (**Supplementary Data File 4**). Interestingly, BLAST results >90% identical to SURF_17 (the generally accepted cutoff for family level lineage) are all from deep subsurface environments, including freshwater aquifers (Flynn et al., 2013), gas hydrates, deep-sea hydrothermal sediments and deep-sea sediments from the Mariana Trough (Kato et al., 2015). Indeed, the only 16S rRNA gene sequence >98% identical to that for SURF_17, the accepted cutoff for same species lineage (Yarza et al., 2014), was collected from the world’s deepest sinkhole in Zacatón, Mexico (Sahl et al., 2010). Considering the global distribution and apparent habitat restriction to subsurface environments, we propose that ‘*Ca.* Abyssubacteria’ is a lineage metabolically suited to dark and often anoxic subsurface environments.

Phylogenomic and amino acid identity analyses indicate that the two SURF genomes analyzed here are a relatively divergent lineage, likely constituting a novel class or order within the proposed candidate phylum, ‘*Ca.* Hydrogenedentes’ (**Figure 1**, **Supplementary Figure 1**, and **Supplementary Data File 4**). To date, only a handful of ‘*Ca.* Hydrogenedentes’ genomes have been sequenced and made publicly available. The first genome that corresponded to the unclassified group ‘NKB19’ according to 16S sequence identity was a compilation of four single cell amplified genomes (SAGs), combined into one genome, identified as Candidatus_Hydrogenedens_terephthalicus_JGI_OTU1 (Rinke et al., 2013) (**Figure 1**, **Supplementary Figure 1**, and **Supplementary Data File 2**). These cells were collected from an anaerobic terephthalate-degrading sludge bioreactor (Rinke et al., 2013). Very recently, two more genomes belonging to this candidate phylum were sequenced, UBA2224 and UBA6118 (Parks et al., 2017). These genomes improved upon the completeness and contamination of the combined SAGs (∼85% complete to ∼98% complete and ∼4% versus ∼1% contaminated, respectively), but no functional analysis was provided for these new MAGs. Here, we perform an in depth functional analysis of the SURF_5 and _17 MAGs and compare and contrast them to the previously sequenced ‘*Ca.* Hydrogenedentes’ genomes: Candidatus Hydrogenedens terephthalicus_JGI_OTU1, UBA2224 and UBA6118.

### Putative Carbon Metabolism

Both SURF_5 and SURF_17 have all the genes necessary for the reductive acetyl-CoA pathway, the only carbon fixation pathway known to be used by both Archaea and Bacteria (Hügler and Sievert, 2011). It also requires the lowest energy input of all the six known carbon fixation pathways (Berg et al., 2010; Hügler and Sievert, 2011), making it ideal for organisms operating in energy-deplete subsurface environments where nutrients have been highly recycled. Furthermore, the reductive acetyl-CoA pathway requires anoxic conditions, as some of its enzymes, especially the crucial acetyl-CoA synthase, are highly oxygen sensitive (Berg et al., 2010). Because of energetic efficiency and the necessity for anoxia, the reductive acetyl-CoA pathway is the ideal mode of inorganic carbon fixation in highly reducing, aphotic and energy-deplete deep subsurface fluids, including those encountered at SURF, where the oxidation-reduction potential was assessed at -235 to -276 mV (Osburn et al., 2014). Interestingly, the SURF genomes are the first members of the ‘*Ca.* Hydrogenedentes’ reported to have carbon fixing capability via the reductive acetyl-CoA pathway (**Figure 3**). The only other *‘Ca.* Hydrogenedentes’ genome having any possibility of carbon fixation is UBA6118, containing the canonical gene for RuBisCo (**Figure 3**). If the ability to fix carbon via the relatively energy inexpensive reductive acetyl-CoA pathway is widespread in the closest relatives of SURF_5 and _17 that appear to be distributed in global subsurface environments (**Figure 4**), they could be important sources of fixed carbon in subsurface fluids that often contain highly recycled and recalcitrant carbon sources.

*‘Ca.* Abyssubacteria’ genomes contain genes for the Embden-Meyerhof glycolysis pathway and the pentose phosphate pathway, which links carbon fixation to biomass and carbohydrate synthesis and enables the generation of glycogen as a storage compound. Additionally, both genomes contain both *pdh-* and *por-*encoded pyruvate dehydrogenase and ferredoxin oxidoreductase genes. These couple the reductive acetyl-CoA pathway to the Embden-Meyerhof glycolysis pathway, and subsequently to the TCA cycle, by catalyzing the oxidation of pyruvate to acetyl-coA or acetate (Ragsdale and Pierce, 2008) (**Figure 2**). Typically, the *por* gene suite is used in acetogens and other anaerobes because these enzymes use low potential electron transfer proteins like ferredoxin and flavodoxin, which likely makes the pyruvate synthase reaction feasible as the first step for converting acetyl-CoA into cell material. It is possible that *‘Ca.* Abyssubacteria’ genomes are expressing *por* genes in subsurface fluids at SURF, rather than genes that encode pyruvate dehydrogenases, because ferredoxin is a low-potential electron donor (Ragsdale and Pierce, 2008). The electron donor for pyruvate dehydrogenases, NADH, requires 200 mV more than the acetyl-CoA/pyruvate couple and thus cannot reduce acetyl-CoA. Given the presence of por genes and a complete reductive acetyl-CoA pathway (which is often used by acetogens) in both SURF genomes, acetogenesis is a possible energy metabolism.

Autotrophic growth using the reductive acetyl-CoA pathway does not produce ATP by substrate-level phosphorylation: it is an energy-requiring process and must be coupled to an exergonic anaerobic respiratory process (Ragsdale and Pierce, 2008). Nitrogen reduction is a possible energy-yielding strategy given both the presence of nitrogen transforming genes in *‘Ca.* Abyssubacteria’ genomes (*nar, nrfA*, and qNOR) and the exergonic Gibbs energy of oxidized nitrogen as an electron acceptor in SURF fluids (Osburn et al., 2014). However, carbon assimilation via the reductive acetyl-CoA pathway is typically blocked in the presence of nitrate (Müller, 2003; Ragsdale and Pierce, 2008). This inhibition leads one to question why *‘Ca.* Abyssubacteria’ genomes have retained all genes involved in the reductive acetyl- CoA pathway. Note that the reductive acetyl-CoA pathway can operate in reverse, with heterotrophs using carbon monoxide dehydrogenase and acetyl-CoA synthase to oxidize acetyl-CoA (Rabus et al., 2006). Hence, it cannot be ruled out that *‘Ca.* Abyssubacteria’ found in SURF fluids could be employing the reductive acetyl-CoA pathway heterotrophically. ‘*Ca.* Abyssubacteria’ genomes contained all requisite genes for methylotrophy, including multiple corrinoid protein methyltransferases, indicating a possible venue for multiple organic carbon based energy metabolisms, including homoacetogenesis, as discussed previously.

We searched for energy-yielding pathways and found evidence for a complex oxidative phosphorylation pathway in both MAGs, which suggests aerobic respiration and the transfer of electrons to molecular oxygen (**Figures 3, 4** and **Supplementary Data File 3**). However, oxygen levels were below detection in subsurface fluids at SURF (Osburn et al., 2014). We searched for further evidence of oxygen respirations, such as cytochrome c oxidases, but could not identify any in either MAG. We think it is more likely that *‘Ca.* Abyssubacteria’ at SURF are using anaerobic metabolisms such as nitrogen reduction, sulfate reduction or sulfite oxidation as their predominant energy metabolisms *in situ*, discussed in more detail below.

### Putative Energy Metabolisms

In denitrification, energy is conserved as nitrate (NO_3_^-^), nitrite (NO_2_^-^), nitric oxide (NO) and nitrous oxide (N_2_O) are sequentially reduced to dinitrogen gas (N_2_), each step catalyzed by one or more metalloenzymes (Mahne and Tiedje, 1995; Zumft, 1997). *‘Ca.* Abyssubacteria’ genomes contained genes (*nar*) encoding enzymes that catalyze the first step of this pathway (**Figure 2**). The first step, the reduction of nitrate to nitrite, is enabled by the transport of NO_3_^-^ into the cell by an ATP-binding cassette (ABC)-type NRT in *‘Ca.* Abyssubacteria’, which putatively provides NO_3_^-^ for reduction by Nar enzymes (**Figure 2**). The NO_2_^-^ produced by this reaction is also a highly reactive possible electron acceptor. In the three publicly available ‘*Ca.* Hydrogenedentes’ genomes (Candidatus_Hydrogenedens_terephthalicus_JGI_OTU1, UBA2224 and UBA6118) it appears that nitrite could be reduced to ammonium via DNRA. The requisite genes for this metabolism are *nrfABEFG*, which are present in Hydrogenedens_terephthalicus_JGI_OTU1, UBA2224 and UBA6118 (**Figure 3**). However, we were only able to identify the gene for the canonical cytochrome c, *nrfA*, in SURF_5 but not in SURF_17 (**Figures 2, 3**). We hypothesize that the other *nrf* genes have been lost in SURF genomes, and instead of DNRA they are likely performing other nitrogen, sulfur or carbon based metabolisms that are more exergonic in the subsurface fluids from which they were collected. Dissimilatory NO reduction to N_2_O can be catalyzed by two classes of the NorB enzyme: a quinol-oxidizing single subunit (qNorB) or a cytochrome *bc-*type multiplex (cNorB) (Zumft, 1997). SURF_5 and SURF_17 possess the quinol-oxidizing nitric oxide reductase, bound to the inner membrane of the cell (**Figure 2**). However, because NO is a highly toxic molecule, interfering with cellular processes, we cannot rule out the possibility that the function of the nitric oxide reductase, qNOR, is for detoxification rather than an energy yielding process. Considering the measured nitrate levels in SURF fluids (10–25 μM, Osburn et al., 2014), it appears likely that *‘Ca.* Abyssubacteria’ could be performing the first step of denitrification in the subsurface at SURF, reduction of nitrate to nitrite. Given the presence of methylotransferase genes in SURF_5 and SURF_17 genomes, it is possible that these bacteria are coupling the anaerobic oxidation of methylated compounds, such as methanol or methylamine, using nitrate as an electron acceptor. Although this metabolic strategy is not widespread, anaerobic methylotrophy coupled to denitrification is exhibited in several marine and wastewater-associated species, including *Methylophaga nitratireducenticrescens*, *Hyphomicrobium denitrificans*, and *Methyloversatilis* spp. (Urakami et al., 1995; Baytshtok et al., 2008; Mauffrey et al., 2017).

Another possibility for energy yielding chemotrophic metabolism in *‘Ca.* Abyssubacteria’ is the dissimilatory oxidation or reduction of sulfur compounds. *‘Ca.* Abyssubacteria*’* genomes contain putative dissimilatory capabilities, with genes for *sat* and *aprAB* possibly oxidizing sulfite to APS and then to sulfate, respectively (**Figure 2**). This pathway can also be run in reverse, catalyzing the stepwise reduction of sulfate to sulfite and then sulfide. However, a genome-wide search revealed only the presence of assimilatory sulfite reductases. Neither the dissimilatory sulfite reductase genes (*dsrAB*), nor the requisite companion gene, *dsrD*, was identified. Therefore, the SURF genomes appear capable of only the first step in sulfate reduction, that of SO_4_^2-^ reduction to SO_3_^-^ via *sat* and *aprAB*, and not further reduction to sulfide via *dsrABD*.

Although both genomes contained the *SoxD* gene, *‘Ca.* Abyssubacteria’ likely cannot oxidize sulfide, as other *Sox* genes necessary to complete the pathway were not present. The presence of heterodisulfide reductases and other reductive enzymes suggests that *‘Ca.* Abyssubacteria’ could be using H_2_ as an electron donor when available *in situ*. However, given that sulfate reduction with H_2_ is only moderately exergonic in SURF fluids, and that sulfate reduction fell near anaerobic heterotrophy in a principal component analysis of thermodynamic favorability using *in situ* fluid geochemistry (Osburn et al., 2014), it is also possible that *‘Ca.* Abyssubacteria’ utilizes organic matter as an electron donor. Given the presence of genes that encode enzymes for hydrogenases and methylotrophy, electron donor utilization appears to be versatile in SURF genomes, is most likely varied depending upon the electron acceptor concentration, and on subsurface geochemical conditions *in situ.* The retention of genes encoding multiple putative methods of carbon, nitrogen and sulfur metabolisms may be a way for *Ca.* Abyssubacteria to cope with variable geochemistry and intermittent energetically unfavorable conditions.

The candidate phylum ‘Hydrogenedentes’ was named for the abundance of hydrogenases and putative H_2_-utilizing pathways in the four SAGs that were the first partial genomes representing the phylum (Rinke et al., 2013). Later, metagenomic and metatranscriptomic analyses of the microbial community in a methanogenic bioreactor identified abundant read mapping to putative ‘*Ca.* Hydrogenedentes’ and identified them as lipolytic glycerol degraders (Nobu et al., 2015). Indeed, to date, all of the available genomes belonging to the ‘*Ca.* Hydrogenedentes’ have been collected from engineered environments such as anaerobic sludge bioreactors (Rinke et al., 2013; Nobu et al., 2015). In terms of 16S rRNA sequences, after the collection of the original ‘NKB19’ from the depths of the Mariana trench, subsequent published 16S sequences identifying with this group have been collected from phthalate-degrading bioreactors (Rinke et al., 2013) and methanogenic sludge bioreactors (Rivière et al., 2009; Narihiro et al., 2015; Nobu et al., 2015). In contrast, the two genomes analyzed here were collected from deep continental subsurface fluids, in a relatively natural and undisturbed environment, compared to anaerobic sludge bioreactors. Their closest relatives, according to the vast repository of 16S rRNA clone sequences, reside in similar continental and marine subsurface environments (**Figure 4** and **Supplementary Data File 4**). This discrepancy in habitat is reflected in the putative metabolisms encoded by available genomes (**Figure 3**). The three genomes that comprise the ‘*Ca.* Hydrogenedentes’ phylum are putative organic carbon degraders, potentially hydrolyzing carbon compounds such as phthalates, lipids and glycerols. In contrast, the genomes from SURF contain all genes necessary for autotrophic carbon fixation via the reductive acetyl-CoA pathway (**Figures 2, 3**). Although genome reconstruction was not possible, Nobu et al. were able to construct a pangenome and metatranscriptome putatively belonging to *Ca.* Hydrogenedentes and found that they were likely metabolizing lipids, hydrolyzing triaglycerols to glycerol and long chain fatty acids, and/or syntrophically oxidizing glycerol to carbon dioxide and acetate (2015). None of these appear to be the most likely metabolisms for the genomes collected from SURF. In our metabolic analysis of all five currently available genomes in the ‘*Ca.* Hydrogenedentes’ phylum, the only energy metabolisms they may have in common are homoacetogenesis and sulfate reduction to sulfite (**Figure 3**).

### Concluding Remarks and Description of ‘*Ca*. Abyssubacteria’

Based on our phylogenomic analysis using a concatenated alignment of single-copy marker genes (**Figure 1**), phylogenetic analysis of 16S rRNA gene sequences (**Supplementary Figure 1** and **Supplementary Data File 4**), and AAI and POCP analyses (**Supplementary Data File 2**), the two MAGs identified in this study are proposed as a novel class or order-level lineage that most likely falls within the candidate phylum “Hydrogenedentes.” We designate this new candidate lineage ‘*Candidatus* Abyssubacteria’, from the Latin prefix meaning deep, owing to their collection 1.5 km below surface. Cells are motile, and have versatile carbon and energy-yielding chemotrophic metabolic potential, enabling them to survive in dark, energy deplete subsurface environments under varying levels of oxygen, nitrogen, carbon, and sulfur.

## Data Deposition

Sequence data for metagenomic reads from SURF, South Dakota, contigs and genes were submitted to the JGI-IMG under accession number IMG 3300007351. Sample metadata for those sequences can be accessed using the BioProject identifier PRJNA355136. Genomes comprising the proposed candidate phylum Abyssubacteria, SURF_5 and SURF_17, can be found on the publicly accessible database, NCBI, under accession numbers SAMN08498999 and SAMN08499011, respectively.

## Author Contributions

LM analyzed the metagenomic data, performed the phylogenetic analyses, and reconstructed the metabolism of genomes analyzed in the study and wrote the manuscript. HA analyzed the global distribution, performed the phylogenomic analyses, POCP calculations and contributed to results and discussion. JA funded sample retrieval and provided guidance during analysis and writing of the manuscript.

## Conflict of Interest Statement

The authors declare that the research was conducted in the absence of any commercial or financial relationships that could be construed as a potential conflict of interest.
