# Supplementary material for: Genomic Description of ‘Candidatus Abyssubacteria,’ a Novel Subsurface Lineage Within the Candidate Phylum Hydrogenedentes
Source: Front Microbiol. 2018 Aug 28;9:1993. doi: 10.3389/fmicb.2018.01993 (PMC6121073; doi:10.3389/fmicb.2018.01993)
Supplement: Supplementary file 1 [file Data_Sheet_1.docx]

**Supplementary Data File 1. List of proteins concatenated**

**and used to build phylogenetic tree in Figure 1.**

| Protein Name |
| --- |
| Ribosomal Protein L2 |
| Ribosomal Protein L3 |
| Ribosomal Protein L4 |
| Ribosomal Protein L5 |
| Ribosomal Protein L6 |
| Ribosomal Protein L14 |
| Ribosomal Protein L15 |
| Ribosomal Protein L16 |
| Ribosomal Protein L18 |
| Ribosomal Protein L22 |
| Ribosomal Protein L24 |
| Ribosomal Protein S3 |
| Ribosomal Protein S8 |
| Ribosomal Protein S10 |
| Ribosomal Protein S17 |
| Ribosomal Protein S19 |
