# Supplementary material for: Genomic Description of ‘Candidatus Abyssubacteria,’ a Novel Subsurface Lineage Within the Candidate Phylum Hydrogenedentes
Source: Front Microbiol. 2018 Aug 28;9:1993. doi: 10.3389/fmicb.2018.01993 (PMC6121073; doi:10.3389/fmicb.2018.01993)
Supplement: Supplementary file 5 [file Image_1.pdf]

Supplementary Figure 1

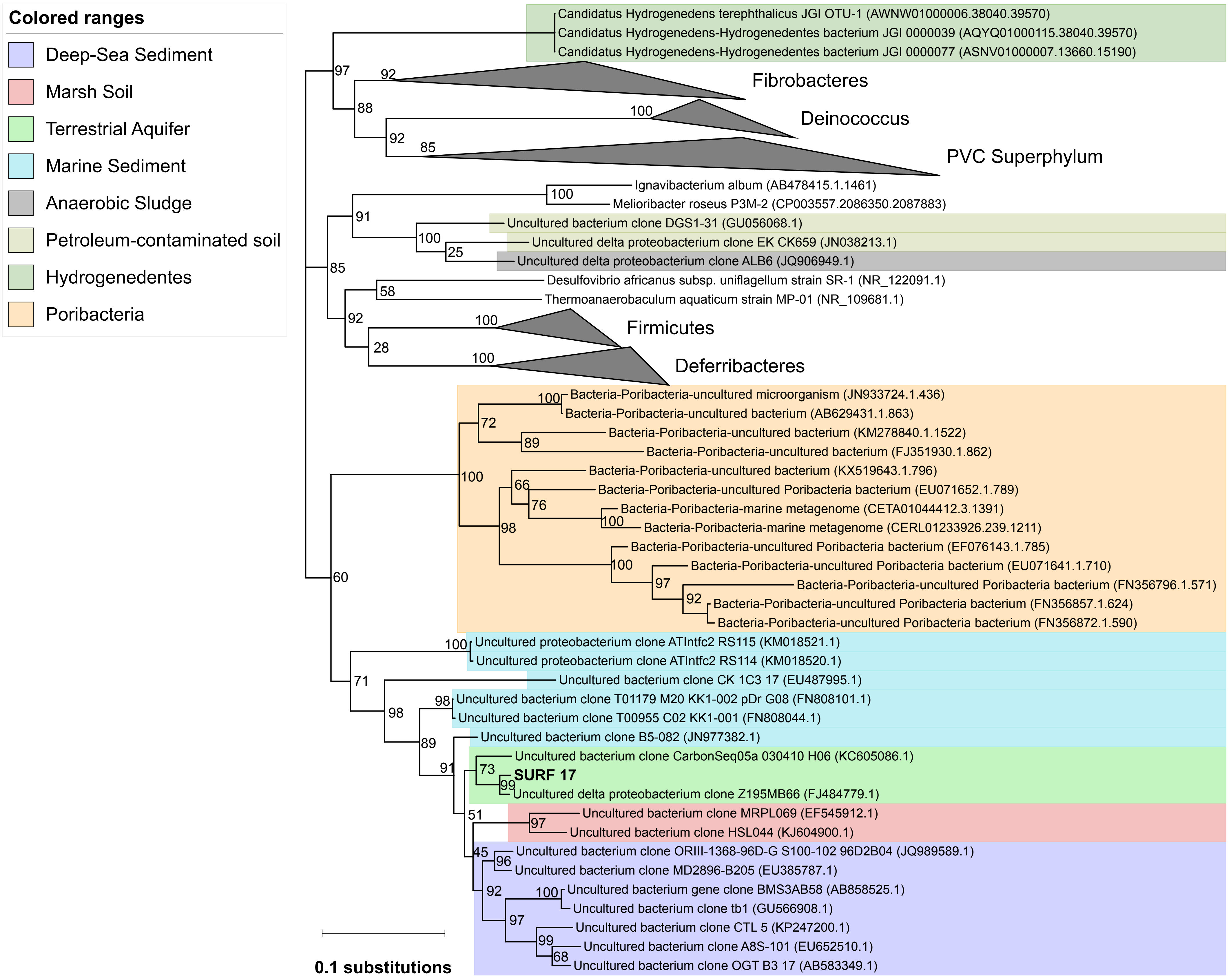

Supplementary Figure 1. 16S rRNA ribosomal protein tree comparing SURF_17 sequence to 16S clone sequences available on the NCBI nr and ref_seq databases. SURF_17 is grouping with uncultured clones from terrestrial aquifers and marine deep sea sediments, separately from the previously named Ca. Hyrogenedentes that were collected from anaerobic bioreactors.
