# Supplementary material for: Genomic Description of ‘Candidatus Abyssubacteria,’ a Novel Subsurface Lineage Within the Candidate Phylum Hydrogenedentes
Source: Front Microbiol. 2018 Aug 28;9:1993. doi: 10.3389/fmicb.2018.01993 (PMC6121073; doi:10.3389/fmicb.2018.01993)
Supplement: Supplementary file 6 [file Image_2.pdf]

Supplementary Figure 2

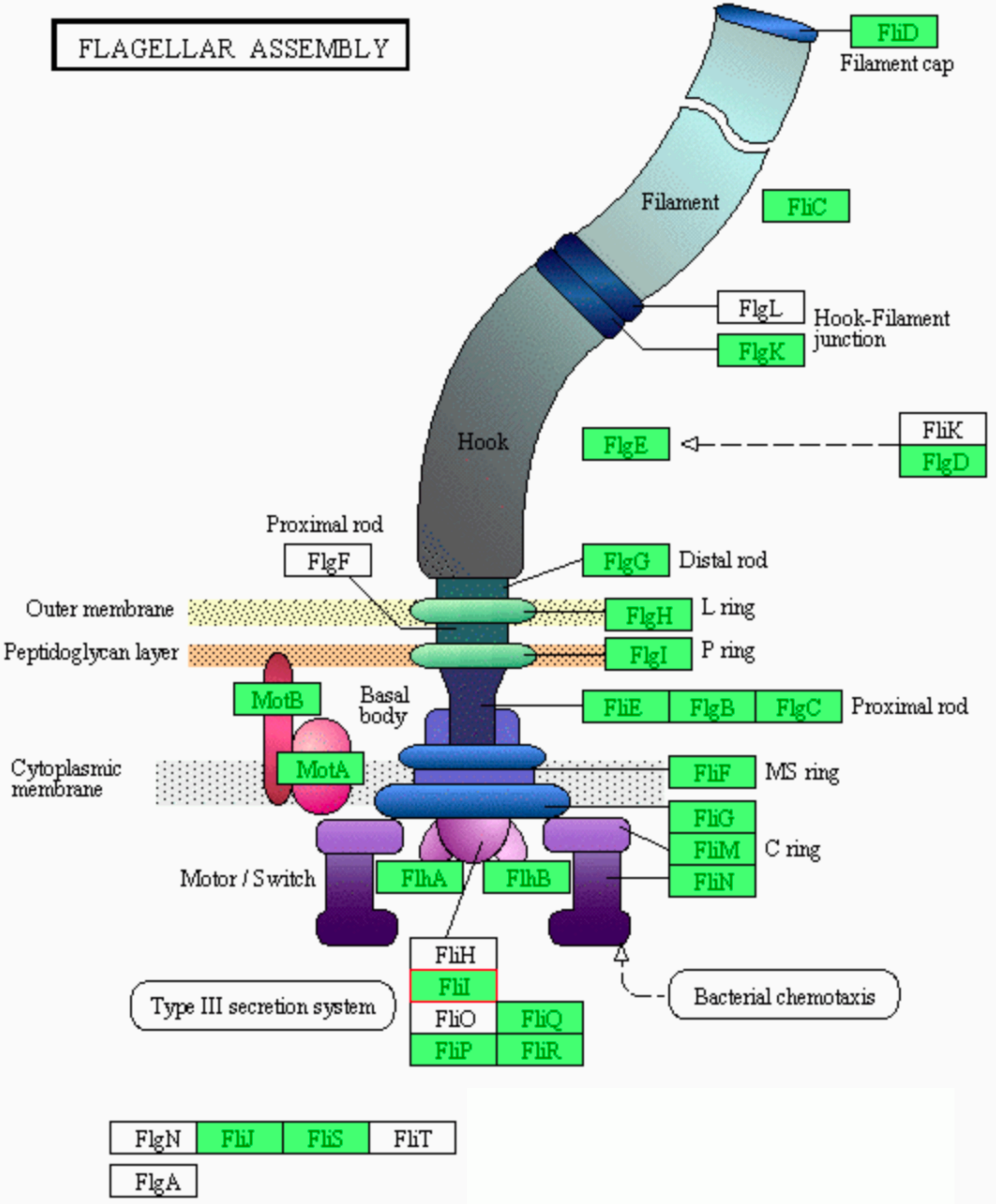

Supplementary Figure 2. Expanded view of the flagellar assembly. Genes encoding relevant proteins that were found in the Ca. Abyssubacteria genomes are highlighted in green. Genes not present are white. Figure generated on the IMG/ER database.
